# Impact of Mixed Infections of Gut Parasites *Lotmaria passim* and *Nosema ceranae* on the Lifespan and Immune-related Biomarkers in *Apis mellifera*

**DOI:** 10.3390/insects11070420

**Published:** 2020-07-08

**Authors:** Nolberto Arismendi, Solange Caro, María Paz Castro, Marisol Vargas, Gustavo Riveros, Tomas Venegas

**Affiliations:** 1 Austral Biotech Research Center, Faculty of Science, Universidad Santo Tomás, Av. Ramón Picarte, Valdivia 1130, Chile; 2 Laboratories of Virology and Bee Pathology, Facultad de Agronomía, Universidad de Concepción, Av. Vicente Méndez, Chillán 595, Chile; solangecaro@udec.cl (S.C.); mcastrot@udec.cl (M.P.C.); marisolvargas@udec.cl (M.V.); gustavoriveros@udec.cl (G.R.); rovenegas@udec.cl (T.V.)

**Keywords:** AMPs, vitellogenin, lifespan, trypanosome, microsporidium, honey bee

## Abstract

*Lotmaria passim* currently appears to be the predominant trypanosome in honey bees worldwide. Although, the specific effects of *L. passim* by single or mixed with other gut parasites such as *Nosema ceranae* on honey bees’ health is still unclear. We consequently measured bees’ survival, parasite loads, the expression of antimicrobial peptides (AMPs) and vitellogenin gene. Thus, (1) bees naturally infected with *L. passim*, (2) healthy bees inoculated with *Nosema ceranae*, (3) bees naturally infected with *L. passim* and inoculated with *N. ceranae* and (4) healthy bees (control) were maintained under controlled conditions. Honey bees infected with *N. ceranae* or with mixed infections of *L. passim* and *N. ceranae* had significantly lower survival rates than the control group at 20 days post-inoculation (dpi). A competitive suppression was also detected, provided that the *L. passim* load was significantly affected by the presence of *N. ceranae* at 15 dpi. Expressions of the AMPs defensin and hymenoptaecin rapidly (two hours post-inoculation) increased in bees infected with *N. ceranae* and mixed infections. However, this effect was not continuous. In fact, expressions of abaecin, defensin, hymenoptaecin and vitellogenin decreased drastically at 15 dpi in bees with both single and mixed infections. The decrease in the expression of AMPs and vitellogenin throughout this period was consistent with the reduced survivals observed in this study, indicating that mixed infections of *L. passim* and *N. ceranae*, and even into a scenario of competition between them, may have a synergic effect on the survival and immune-related gene expressions (biomarkers) of worker bees.

## 1. Introduction

There are currently two species of trypanosomes that invade the gut of *Apis mellifera* (L.); *Crithidia mellificae* Langridge and McGhee and *Lotmaria passim* Evans and Schwarz. *Lotmaria passim* was described only a few years ago [1] and is now considered the most predominant trypanosome of honey bees in Asia, Europe and South and North Americas, wherein *C. mellificae* is considered infrequent or absent [1,2,3,4]. Trypanosomes in bee species have become more relevant in recent years, since they have proven to have negative effects on behavior, physiology, the immune system and the lifespan of the hosts [5,6,7,8]. However, the specific effects of *L. passim* on honey bee survival, immunity and host physiology is still unclear. On the other hand, the gut parasite *Nosema ceranae* Fries (Microsporidia) has been more thoroughly considered as a factor contributing to bee losses, either alone or in combination with other parasites [9,10,11,12]. *Nosema ceranae* was first reported in European honey bees around 10 years ago in Europe and Asia [13,14]. Since these first reports, *N. ceranae* has now become one of the most prevalent honey bee microsporidia worldwide [15]. This parasite has been implicated in honey bee immunosuppression, the reduction of the host lifespan and physiological and behavioral changes, as well as negatively affecting the productivity of honey bee colonies [10,16,17,18]. *Nosema ceranae* has also been implicated in the global phenomenon of colony loss, at least in Spain [9].

It is not unusual that multiple parasite genotypes, either distinct strains of the same species or different species, coinfect a single host [19], especially in social insects, where the transmission of beneficial or pathogen agents may be facilitated by social interactions [20]. Pathogen mixed infections may interact in different ways within the host; in some cases, pathogens can act independently of one another; positive interactions are also possible, when one proliferates due to the presence of the other, and finally, negative interactions can also occur when pathogens suppress one another [21]. Experimental evidence has shown a synergistic effect when mixed infections of *N. ceranae* and other honey bee pathogens have been observed [12]. These mixed infections may alter local and systemic immune gene transcriptions, the survival and, in some cases, the learning and memory capabilities of honey bees [7,11,22]. However, there is no experimental evidence related to how a mixed infection of *N. ceranae* and *L. passim* could affect honey bee colonies. Some current reports have shown that mixed infections of these pathogens are not common (no more than 2%), in spite of the fact that they have been detected in high prevalence in single infections in honey bee hives [3,23], suggesting a possible competition between these two parasites. Furthermore, such mixed-species infections may influence the host immune responses, including genes that elicit antimicrobial peptides (AMPs) [7,16]. AMPs are considered as a key component in insect innate immunity, and four families of AMPs (abaecin, apidaecin, defensin and hymenoptaecin) have been described in honey bees [24,25]. There is evidence that honey bee immune responses (AMPs) are dynamic in transcriptional intensity and diversity over short or long periods of time—for instance, after *N. ceranae* infections [7,16]. Furthermore, single and mixed infections of parasites may also affect other relevant components associated with immune senescence, such as the phospholipoglycoprotein vitellogenin [26]. In worker honey bees, vitellogenin play multiple roles; it is important in bee behaviors associated with nursing and foraging activities but is also essential for bees’ immune responses, oxidative stress resilience (antioxidant properties) and longevity [27,28,29]. Therefore, AMPs and vitellogenin genes could be used as biomarkers for honey bee health and physiology, since their expression can be affected by biological stressors such as varroa mites, microsporidia, trypanosomes and viruses [16,30,31,32]. New research is thus required in order to determine the effects of *L. passim* alone or in combination with *N. ceranae* on honey bees’ health and, also, to determine whether the interaction between these two gut pathogens has a synergistic effect, a competitive effect or no effect at all. To address the possible impacts of single and mixed infections of these two gut parasites on honey bees’ lifespans and their expression of AMPs and the vitellogenin gene (Vg), we monitored the signaling of AMPs and oxidative stress related-gene (Vg) expressions over a 20-day period post-inoculation. We also quantified the parasite loads and bee survival rates of honey bees with a basal level of *L. passim* infection, as well as bees (both healthy and *L. passim*-infected) that were inoculated with *N. ceranae* spores after emergence.

## 2. Materials and Methods

### 2.1. Biological Assays in Honey Bees

Provided that we have detected *L. passim* in honey bee workers of different ages and developmental stages [33] and, also, the difficulty to obtain pure cultures of *L. passim* cells, we decided to evaluate the possible effects of *N. ceranae* in bees from colonies with a basal level (1.0 ± 0.6 × 10^3^ cell per bee) of *L. passim* in addition to uninfected honey bees. Furthermore, this experiment gave the opportunity to evaluate the effects of the occasional infection of *N. ceranae* in bees that were previously infected with *L. passim*. Additionally, it is necessary to mention that, at the beginning of the season (spring), all the queens in the colonies were replaced by new queens (*A. mellifera carnica*) that were bought at the same honey bee queen supplier company (https://www.jphmielapicultura.cl/) in order to ensure similar queen genetics in the colonies and, thus, to avoid a genetic effects of the queen on worker bees used in the experiment.

The health status of colonies (n = 40) was previously determined by molecular techniques according to Vargas et al. [3] (Table S1, Supplementary Materials). Then, brood combs with capped worker bees were removed from uninfected (n = 1 per colony, 4 colonies) and *L. passim*-infected colonies (n = 1 per colony, 4 colonies) and maintained under controlled conditions in a rearing room (30 °C ± 1.0 and 60% ± 3.0% relative humidity (RH)). Afterwards, newly emerged worker bees were carefully collected from the brood comb and randomly confined in plastic cages (base = 8 cm diameter, mouth = 10 cm diameter and height = 15 cm) to reduce the effects of the colony genetics on the experiments. The bees were supplied with sucrose syrup 60% (w/v) for 24 h. After this time, the live bees were used for assays. Therefore, to test the effects of *L. passim* alone or its synergistic effects due to a mixed infection with *N. ceranae*, the treatments were as follows: (1) bees naturally infected with *L. passim* (Lp), (2) healthy bees inoculated with *N. ceranae* (Nc), (3) bees naturally infected with *L. passim* and inoculated with *N. ceranae* (mixed-species infection) and (4) uninfected honey bees (control treatment). In the bees that had to be inoculated with *N. ceranae*, the spores were purified from *N. ceranae*-infected colonies according to the methodology described by Fries et al. [34]. Thus, the bees were starved for 3 h and were then individually infected with *N. ceranae* spores (1.0 ± 0.3 × 10^5^ spores per bee), which were mixed in 60% sucrose syrup (5 μL per bee) according to Porrini et al. [35]. Those bees that did not consume the total amount of *N. ceranae* spore suspensions were discarded from the assay. Then, 75 worker bees (2 days old) were maintained in plastic cages (4 replicates per treatment) containing 3 g of pollen substitute (soybean meal (18%), curbiculated pollen (10%), corn flour (6%), wheat flour (6%), potato starch (2%), canola oil (0.2%) and sucrose (57.8%) mixed in distilled water to make a patty) and supplied with 60% sucrose syrup ad libitum [36]. Dead bees were counted daily and removed from cages until the end of the experiment (20 days total) for a survival analysis. Furthermore, five bees were removed from each cage after 2 h, as well as 5, 10 and 15 days post-inoculation (dpi) in all treatments for a molecular analysis.

### 2.2. Nucleic Acid Extraction, cDNA Synthesis and Real-Time PCR

Honey bee abdomen samples (5 abdomens per replicate) were homogenized with 5 mL of phosphate-buffered saline (PBS 1X) with a mortar and pestle in cold conditions. Then, 200 μL of the abdomen extract was used for RNA extraction according to the instructions provided by the E.Z.N.A. *Total RNA Kit* I (OMEGA, Bio-Tek Inc., Atlanta, GA, USA). However, the TRK lysis buffer included in the RNA Kit I was replaced by 500 μL of Trizol™ Reagent (Invitrogen, Thermo Fisher Scientific, Waltham, MA, USA) in order to improve the RNA isolation.

For the DNA extraction, another 200 μL of the abdomen sample extract was ground in 1.5-mL microcentrifuge tubes using sterile plastic pestles with 350-μL control (CTL) buffer following the E.Z.N.A. Insect DNA Kit’s instructions (Omega, Bio-Tek, Norcross, GA, USA). DNA and RNA were then quantified (Infinite 200 PRO NanoQuant, Tecan Group Ltd., Männedorf, Switzerland) and stored at −80 °C.

The RNA extracted was used for first-strand cDNA synthesis, which was performed using the M-MLV reverse-transcriptase enzyme (Invitrogen, Life Technologies, Carlsbad, CA, USA), according to the manufacturer’s instructions. The cDNA samples were maintained at −20 °C until the real-time PCR (qPCR) analyses were carried out.

In order to quantify the *L. passim* and *N. ceranae* loads in infected samples (with both single and mixed-species infections), a real-time PCR (qPCR) was carried out with specific primers capable of amplifying the ribosomal RNA gene in both gut parasite species (Table 1). Additionally, to detect and quantify the possible changes in the expression of the immune-related genes that encoded AMPs such as abaecin, defensin (defensin-1) and hymenoptaecin, as well as vitellogenin (Vg), we used primers that have been previously published (Table 1). Thus, the PCR reactions were carried out in 15 μL (4 biological replicates per treatment and two technical replicate per sample), containing 20 ng of cDNA, 1X of KAPA SYBR FAST Universal 2X qPCR Master Mix (Kapa Biosystems, Wilmington, MA, USA), 530 nM of forward primer, 530 nM of reverse primer and enough sterile-filtered molecular grade water to reach the total 15 μL. The thermal conditions were achieved with one cycle at 95 °C for 10 min, followed by 40 cycles at 95 °C for 15 s, 60 °C for 15 s and 72 °C for 15 s. A dissociation analysis was conducted after all of the amplifications were completed in order to detect the primer dimmers and the unspecific amplicons. Thus, the data regarding the pathogen loads were reported as cell or spore equivalents per bee, according to Tritschler et al. [23]. Similarly, the data concerning immune-related genes were reported as relative expressions after normalization with an endogen gene (β-actin), according to Pfaffl [37].

### 2.3. Data Analysis

Survival curves of uninfected worker bees, as well as those with single and mixed-species infections were plotted using a Kaplan–Meier estimator considering the live bees at the end of the experiment as censored data. Differences among the survival curves were estimated using a Log-rank test (*p* < 0.05), and *p*-values were corrected (*p*′ < 0.05) with the Holm-Bonferroni method [40] for pairwise multiple comparisons with respect to the control group (uninfected bees).

The *L. passim* and *N. ceranae* load data (log_10_) in infected honey bees were not normally distributed (Shapiro–Wilk test, *p* < 0.05). Thus, a Mann-Whitney U test (*p* < 0.05) was run to compare the pathogen loads for each time periods (2 h and 5, 10 and 15 d) post-inoculation. Control treatments (uninfected bees) were excluded from these analyses because no *N. ceranae* or *L. passim* infections were detected in the qPCR.

Statistical differences in the relative expression (log_10_) of AMPs and vitellogenin for each time period post-inoculation for single, mixed-species-infected and uninfected honey bees were estimated by one-way ANOVA. Then, a Dunnett test was run to separate the means in *L. passim* or *N. ceranae*-infected and mixed-species-infected bees with respect to the control. All analyses were carried out with STATISTICA 7.0 software (StatSoft, Tulsa, OK, USA).

## 3. Results

**Figure 1:**
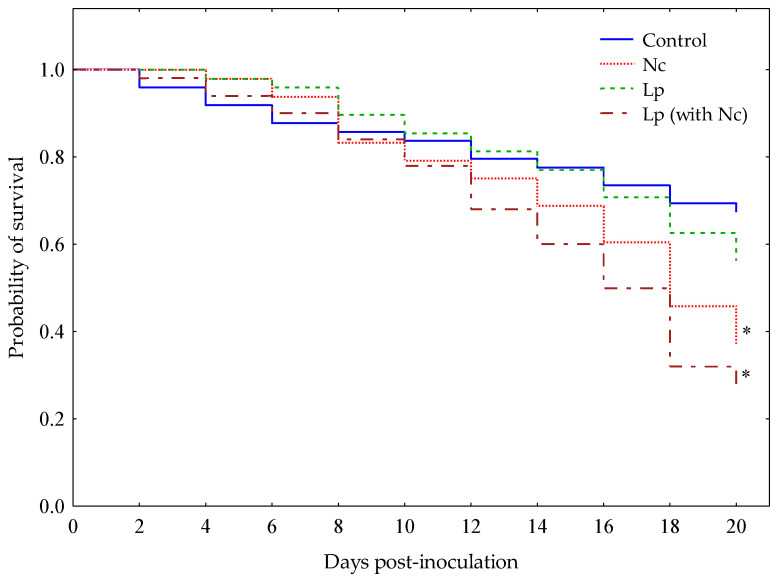
Kaplan-Meier survival curves distribution of worker bees in each experimental group during the 20 days. Each curve represents bees naturally infected with *Lotmaria passim* (Lp), healthy bees inoculated with *N. ceranae* (Nc), bees naturally infected with *L. passim* and inoculated with *N. ceranae* (Lp (with Nc) = mixed-species infection) and uninfected honey bees (control). Asterisks indicate significant differences (* = *p* < 0.05) of pathogen-infected bees with respect to the control group, according to the Log-rank test with Holm-Bonferroni correction.

Worker bees infected with *N. ceranae* or bees naturally infected with *L. passim* and inoculated with *N. ceranae* (bees with mixed-infections) survived for significantly less time than the control group (uninfected bees) (Nc vs. control, Log-rank test value = 2.52, *p* = 0.012 (*p*′ = 0.038) and Lp (with Nc) vs. control Log-rank test value = 3.57, *p* = 0.003 (*p*′ = 0.010)). In fact, bees infected with *N. ceranae* and bees infected with both *L. passim* and *N. ceranae* showed survivals circa 40% and 30%, respectively, whilst the control group showed a survival over 65% (Figure 1). On the contrary, there was no significant difference (Lp vs. control, Log-rank test value = 0.88, *p* = 0.381) in the survival curve of bees that were naturally infected with *L. passim* and the control at the end of experiment (20 dpi; Figure 1).

**Figure 2:**
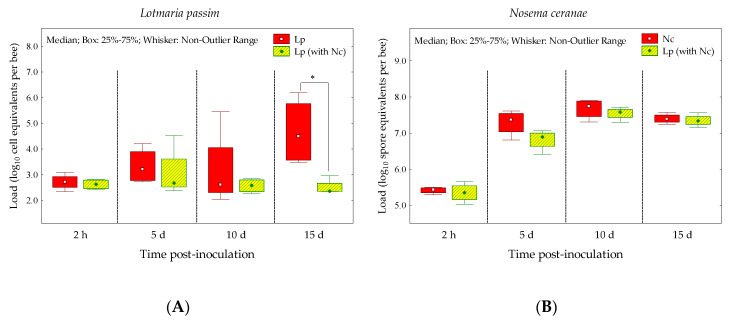
*Lotmaria passim* (**A**) and *N. ceranae* (**B**) loads quantified in bees naturally infected with *L. passim* (Lp) and bees that were *L. passim*-infected and inoculated with *N. ceranae* (Lp (with Nc)) at different times post-inoculation. Asterisks in the box plots indicate significant differences (* = *p* < 0.05) of pathogen loads according to the Mann-Whitney U test.

The *L. passim* load in naturally infected honey bees was significantly affected (Mann-Whitney test U = 0.00, z = 2.31, *p* = 0.021) by the presence of *N. ceranae* after 15 dpi; previous to this time, no differences were observed (Figure 2A). On the other hand, the *N. ceranae* load was not altered by the presence of *L. passim* in the same host (Figure 2B).

**Figure 3:**
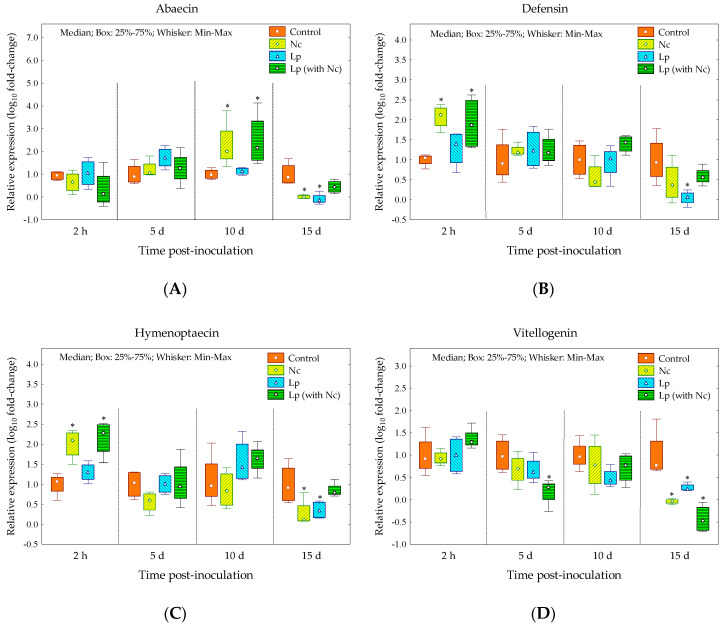
Box plot graphics showing the expressions (relative expressions) of genes encoding the antimicrobial peptides abaecin (**A**), defensin (**B**), hymenoptaecin (**C**) and vitellogenin (Vg) (**D**) in bees naturally infected with *L. passim* (Lp), healthy bees inoculated with *N. ceranae* (Nc), bees that were *L. passim*-infected and inoculated with *N. ceranae* (Lp (with Nc)) and uninfected honey bees (control). Asterisks in the box plot indicate significant differences (* = *p* < 0.05) with respect to the control group, according to the post hoc Dunnett test for each time post-inoculation (2 h, as well as 5, 10 and 15 days).

Honey bees infected with *N. ceranae*, *L. passim* and those mixed-infected with both pathogens induced significant changes in their relative expressions of genes encoding the abaecin peptide 10 and 15 days post-inoculation (ANOVA 10 dpi F_(3,12)_ = 2.34, *p* = 0.047 and 15 dpi F_(3,12)_ = 9.77, *p* = 0.002). Thus, bees infected with *N. ceranae* and those with mixed infections of *L. passim* and *N. ceranae* had significantly higher abaecin mRNA expressions at 10 dpi compared with the control group. However, the abaecin gene expression was significantly downregulated at 15 dpi in single infections of either *L. passim* or *N. ceranae* (Figure 3A). The relative expressions of the genes encoding defensin and hymenoptaecin peptides were also altered significantly in infected bees at 2 h (ANOVA defensin F_(3,12)_ = 5.27, *p* = 0.015 and hymenoptaecin F_(3,12)_ = 10.09, *p* = 0.001) and 15 dpi (ANOVA defensin F_(3,12)_ = 3.53, *p* = 0.049 and hymenoptaecin F_(3,12)_ = 4.43, *p* = 0.026). Defensin and hymenoptaecin mRNA expressions rapidly (2 h post-inoculation (hpi)) increased in bees that were *N. ceranae*-infected and mixed-infected with *L. passim* and *N. ceranae*. Following 2 hpi, no changes in the expressions of either of the aforementioned AMPs were observed at 5 and 10 dpi compared with the control group (Figure 3B,C). Nonetheless, a minor yet significant expression of hymenoptaecin was detected at 15 dpi in *L. passim*-infected and *N. ceranae*-infected bees (Figure 3C). In the case of Vg gene expression levels, significant differences among the analyzed groups were also found at 5 and 15 dpi (ANOVA 5 dpi F_(3,12)_ = 4.07, *p* = 0.033 and 15 dpi F_(3,12)_ = 14.35, *p* < 0.001). The Vg relative expression was significantly lower in bees with mixed infections of *L. passim* and *N. ceranae* compared with the control group at 5 dpi. This effect was not observed to be significant at 10 dpi, but five days later, bees infected with *L. passim*, *N. ceranae* and those with both pathogens showed significant reductions in their levels of the Vg gene expression with respect to uninfected bees (Figure 3D).

## 4. Discussion

To the best of our knowledge, this is the first study to address the effects of infections of *L. passim*, *N. ceranae* and mixed infections of both pathogens considering a basal level of *L. passim* that bees acquired during their development in infected hives. We thus demonstrated, for the first time under controlled conditions, that mixed infections of *L. passim* and *N. ceranae* reduced bee survivals, even more so than those bees that were infected with only *N. ceranae* (Figure 1). This suggests that the interactions produced by mixed infections of both of these pathogens have detrimental effects on bee survivals. On the other hand, survival was not significantly affected in *L. passim*-infected bees. However, the distribution of the survival curve of *L. passim*-infected bees began to drop below that of the control group only after 15–18 dpi (Figure 1), a time period in which the *L. passim* load was higher than the previous measures (Figure 2A). These results indicate that *L. passim* does not induce a rapid death of the host compared with *N. ceranae*, although this assertion should be taken with caution, considering that the initial levels of *L. passim* in infected bees were lower (1.0 ± 0.6 × 10^3^ cell per bee) than the levels of *N. ceranae* (1.0 ± 0.3 × 10^5^ spores per bee) that healthy bees were inoculated with. However, our results were similar to a report by Liu et al. [41], who found that the accumulation of *L. passim* in the hindgut slightly decreased the honey bees’ survival but never resulted in rapid death.

We have observed that the prevalence and loads of these pathogens vary throughout time, especially those of *L. passim*, which remains at low-media levels in spring-summer (1.0 × 10^3^ to 1.0 × 10^5^ cell equivalents per bee) and increases to high levels in fall-winter (3.0 × 10^4^ to 3.0 × 10^11^ cell equivalents per bee) (unpublished data). We also tested the effects of sporadic infections of *N. ceranae* in honey bees infected with a basal level of *L. passim*, considering that this could be a phenomenon that occurs in the field. Based on this hypothesis, we observed a competitive suppression of the *L. passim* load by *N. ceranae*, even when *L. passim* was already established prior to the arrival (inoculation) of *N. ceranae* (Figure 2A). This response could be explained by the fact that the initial infection levels of these pathogens were different, as was stated above. Nonetheless, this competitive effect proved to be significant only after 15 dpi (Figure 2A); before this period, no suppression effect by *N. ceranae* was detected. Competitive suppression by *N. ceranae* is generally expected when a mixed infection occurs in the same space; thus, the pathogens may compete directly for host resources or space in the midgut, as has been reported with *N. apis* [42]. However, the suppression effect of *N. ceranae* on *L. passim* is interesting, considering that these two parasites inhabit different niches within the host. While *N. ceranae* invades and develops in the midgut [43] where its spores are released, these are then accumulated in the hindgut lumen [38]. On the other hand, *L. passim* is normally found in the hindgut, wherein the pathogen cells adhere to the hindgut wall, particularly among the rectal papillae (anterior rectum) and into the lower ileum [1]. Therefore, *N. ceranae* spore accumulations in the hindgut may alter the development of *L. passim* in this gut section, driving a possible competition at least for space. These results could explain, in part, the low prevalence (circa 2%) of mixed infections of *L. passim* and *N. ceranae*, considering that, separately, they have shown a high prevalence in field samples carried out in, e.g., Chile and Switzerland [3,23].

We also demonstrated that honey bee innate immune systems, reflected by the genes encoding AMPs (e.g., defensin and hymenoptaecin), were activated immediately after becoming infected with *N. ceranae*; this was not the case in bees that were naturally infected with *L. passim*. However, this initial increased immune response appeared to be suppressed later not only in bees that were infected by one single species, such as *N. ceranae*, but, also, with *L. passim* (Figure 3). The roles of the AMPs on worker bees infected with *N. ceranae* have already been evaluated in previous studies, generating mixed results [7,16,17,44]. Some studies have shown the activation of gene signals encoding antimicrobial peptides, such as abaecin, defensin and hymenoptaecin, which act against *N. ceranae* infections, especially in the early stages of infection, between a few hours and a few days post-inoculation, inducing an overexpression of these associated genes [7,44]. Nonetheless, immune responses could also be suppressed once *N. ceranae* reaches a significant level of infection within the host [16,17]. We found that, at least in the cases of defensin and hymenoptaecin, gene expressions were activated in the first two hours post the oral inoculation of *N. ceranae* in healthy bees and those with *L. passim*. Nevertheless, we believe that the activation of defensin and hymenoptaecin gene expressions in mixed infections was not due to the combined effects of *L. passim* and *N. ceranae* but, rather, only due to the effect of *N. ceranae* alone, considering that single infections of *L. passim* did not cause a change in these expressions (Figure 3B,C). This quick response of defensin and hymenoptaecin (2 hpi) against the *N. ceranae* infection is an interesting result, considering that no structural development of *N. ceranae* has been observed to occur until 16 h after an inoculation [45]. Schwarz and Evans [7] have found similar results, also detecting changes in the expressions of defensin and hymenoptaecin in the early stages of an infection (e.g., 6 hpi) after an oral inoculation with *N. ceranae* spores. Therefore, it is possible that *N. ceranae* spore components could be delivered to the gut medium immediately after an inoculation, which could then elicit the honey bees’ responses. These components could be effectors (effector proteins), which are secreted by pathogens in order to enter the host, establish a parasitic relationship, survive within the host’s cells and protect the pathogen from the host’s defense mechanisms [46,47]. Some *N. ceranae* effector candidates have been identified, and it has been hypothesized that these effectors play an important role in the *N. ceranae* infection process and its ability to survive within host cells [48]. There is evidence that three days after the initial host cell invasion, *N. ceranae* starts to replicate ventricular cells, releasing newly infectious spores [45,49] that reach a significant infection level at 7 dpi [49]; at this point, the immune responses of the host could be suppressed [16,17]. We observed this phenomenon in the expression of AMPs but only at 15 dpi; no significant changes were found at 5 or 10 dpi (Figure 3). Similar responses were observed in bees that were naturally infected with *L. passim*, wherein the responses of three AMPs were downregulated at 15 dpi. At this point in time, *L. passim* reached a considerable load level (1.0 × 10^6^ cells equivalents per bee; Figure 2A), which suggests that a low load of this trypanosome does not induce changes in AMPs profiles, or it is not detected by honey bee immunity AMPs-dependents, e.g., 5 or 10 dpi. However, in bees infected with *C. mellificae* (strain ATCC 30254), Schwarz and Evans [7] reported that some specific immune-related gene (e.g., *Dscam* and *nimC1*) responses may be downregulated over time, and tissue locations during the course of infection by this type of downregulation were not observed in the expression of the AMPs. This suggests that changes in AMP profiles could be associated with the trypanosome species’ virulence, just as it has been demonstrated with *Nosema* species [16,17]. Additionally, we also expected a downregulation of AMPs in bees with mixed-species infections, considering that multiple pathogen infections may negatively affect the immune systems of the honey bees [7]; however, this response was not significant compared to the control. Therefore, mixed infections of *L. passim* and *N. ceranae* did not necessarily downregulate the expressions of AMPs; this could be due to the competition between the pathogens, which could reduce the cellular infection capabilities and, thus, stimulate the immune response of the host. However, new studies are required to test this hypothesis, including, e.g., an equal initial inoculum of both pathogens at the same and differently sequenced times in order to more precisely measure AMPs and other immune-associated gene responses.

One of the most interesting results was the decreased expression of the Vg gene in all three types of infected bees (including those with only *L. passim*, only *N. ceranae* and mixed-species infections), especially at 15 dpi (Figure 3D). In this study, the suppressed Vg expression in this period was consistent with the breakdown point of the lifespan decline of infected honey bees, especially in bees infected with *N. ceranae* and mixed-species infections (Figure 1). We also observed that the Vg expression was significantly suppressed by mixed-species infections of *L. passim* and *N. ceranae* 5 dpi. This Vg suppression seemed to continue at 10 dpi, although not significantly with respect to the control group. Vitellogenin is a phospholipoglycoprotein that has multiple roles in honey bee health [29]. For instance, vitellogenin can reduce oxidative stress by scavenging for free radicals, thereby prolonging the lifespans of worker and queen bees [28]. This antioxidant activity of vitellogenin is essential to reduce the immune senescence effect on honey bees [26]. Therefore, a suppression of Vg expression could induce rapid cellular senescence, leading to a shortened lifespan in worker bees, an outcome that was observed in this study, especially in bees infected with *N. ceranae* and mixed-species infections.

The differential responses found in this study of genes encoding AMPs and vitellogenin against *N. ceranae* infections, especially over the time of the study and compared with other previously reported results under controlled conditions [7,16,17], could be associated with *N. ceranae* genetic variability [50], the host genotype [51] or spore doses [31]. Even the availability and quality (pollen quality) of nutritional sources could cause interference with bees’ responses to *N. ceranae* infections [52] when bees used in bioassays come from well-nourished colonies. In contrast, the impacts of *L. passim* on AMPs and Vg expressions in infected bees were almost unknown until now. For the first time, we showed that *L. passim* may affect honey bee health, including effects on the immunity and bee survival, at least when it is mixed with other gut parasites. Notwithstanding this, the effects of *L. passim* infections on honey bee health at an individual and colony level, and in association with viral diseases and varroa mite parasites, are still unclear. Therefore, new studies are required to understand the dynamics of this trypanosome over the course of time and under different field conditions, with the aim of determining whether *L. passim* could be a newly contributing factor to colony losses in Chile and around the world.

## 5. Conclusions

In summary, we found a competitive suppression between gut parasites since the *L. passim* load was significantly affected by the presence of *N. ceranae*. However, honey bees infected with *N. ceranae* or with mixed infections of *L. passim* and *N. ceranae* had significantly lower survival rates than the control group. Also, the expression of the AMPs (abaecin, defensin and hymenoptaecin) and vitellogenin decreased drastically in bees with both single and mixed infections and when these pathogens reach high levels. The decrease in the expression of AMPs and vitellogenin throughout this period was consistent with the reduced survivals observed in this study, indicating that mixed infections of *L. passim* and *N. ceranae,* and even into a scenario of competition between them, may have a synergic effect on the survival and immune-related gene expressions of worker bees.

## Figures and Tables

**Table 1:** List of gene-specific primers used in the real-time PCR (qPCR) analysis.

Primer Name	Sequences (5′-3′)	Gene	Size (bp)	Efficiency (%)	Target	References
Lp5F	GGCGTCCGTGATTTTTACTGTGACTA	SSU rRNA	186	103	*Lotmaria passim*	This study ^1^
Lp5R	ACCACAAGAGTACGGAATGCGAAAG					
Nc841F	GAGAGAACGGTTTTTTGTTTGAGA	SSU rRNA	147	102	*Nosema ceranae*	[38]
Nc980R	ATCCTTTCCTTCCTACACTGATTG					
Abaecin-F	CAGCATTCGCATACGTACCA	Abaecin	130	100	AMP abaecin	[24]
Abaecin-R	GACCAGGAAACGTTGGAAAC					
Defensin-F	TGTCGGCCTTCTCTTCATGG	Defensin-1	201	96	AMP defensin	[39]
Defensin-R	TGACCTCCAGCTTTACCCAAA					
Hymenopt-F	CTCTTCTGTGCCGTTGCATA	Hymenoptaecin	200	90	AMP hymenoptaecin	[24]
Hymenopt-R	GCGTCTCCTGTCATTCCATT					
VgMC-F	AGTTCCGACCGACGACGA	Vg	63	94	Vitellogenin precursor	[18]
VgMC-R	TTCCCTCCCACGGAGTCC					
B-actin-F	ATGCCAACACTGTCCTTTCTGG	β-actin	151	96	β-actin (reference gene)	[39]
B-actin-R	GACCCACCAATCCATACGGA					

^1^ New primers used for *Lotmaria passim* detection and quantification were designed and validated according to the procedure described by Arismendi et al. [2]. AMP: antimicrobial peptides.

## Supplementary Materials

The following are available online at https://www.mdpi.com/2075-4450/11/7/420/s1, Table S1: The health status of colonies before the experimental study in laboratory conditions.
